# Visualizing the bidirectional optical transfer function for near-field enhancement in waveguide coupled plasmonic transducers

**DOI:** 10.1038/s41598-018-24061-3

**Published:** 2018-04-10

**Authors:** Lauren M. Otto, D. Frank Ogletree, Shaul Aloni, Matteo Staffaroni, Barry C. Stipe, Aeron T. Hammack

**Affiliations:** 1 Electrical and Computer Engineering, University of Minnesota, Minneapolis, MN USA; 2 HGST (Western Digital Corporation), San Jose & Fremont, CA USA; 3 Molecular Foundry, Lawrence Berkeley National Laboratory, Berkeley, CA USA

## Abstract

We report visualizations of the bidirectional near-field optical transfer function for a waveguide-coupled plasmonic transducer as a metrology technique essential for successful development for mass-fabricated near-field devices. Plasmonic devices have revolutionized the observation of nanoscale phenomena, enabling optical excitation and readout from nanoscale regions of fabricated devices instead of as limited by optical diffraction. Visualizations of the plasmonic transducer modes were acquired both by local near-field excitation of the antenna on the front facet of a waveguide using the focused electron beam of a scanning electron microscope as a probe of the near-field cathodoluminescence during far-field collection from the back facet of the waveguide, and by local mapping of the optical near-field for the same antenna design using scattering scanning near-field optical microscopy as a probe of the near-field optical mode density for far-field light focused into the back facet of the waveguide. Strong agreement between both measurement types and numerical modeling was observed, indicating that the method enables crucial metrological comparisons of as fabricated device performance to as-modeled device expectations for heat-assisted magnetic recording heads, which can be extended to successful development of future near-field-on-chip devices such as optical processor interconnects.

## Introduction

Devices at the frontiers of nanoscience are incorporating light using optical, photonic, and plasmonic elements. While this new scale for engineering light leads to new applications in silicon photonics^1^, sensing and imaging^2^, and data storage^3,4^, it also requires new methods of characterization for device development and optimization. In one of the largest modern efforts for nanoscale optical engineering, the coupling behavior between laser diodes, photonic waveguides, and plasmonic structures is critical to the functioning of heat-assisted magnetic recording (HAMR) in next-generation hard disk drives (HDDs)^3–6^, which remain the primary storage mechanism for the world’s increasing data storage needs (*e*.*g*. data centers or “the cloud”). Determining the bidirectional transfer function for the required near-field optical transducers is a powerful strategy to more fully understand the mechanisms and behavior of photonic waveguides coupled with plasmonic antennas when evaluating different materials for optimal device performance. When illuminated with resonant far-field laser light, plasmonic antennas can generate intense and deep sub-wavelength evanescent near-fields. These plasmonic devices can also be characterized in reverse by exciting plasmonic antennas with focused electron beams, which generate local sub-wavelength optical excitations through cathodoluminescence (CL) that then radiate into the far-field or couple into the modes of neighboring optical waveguides. Bidirectional observation of waveguide-couple plasmonic antennas can offer unique insight into device function and performance.

**Figure 1:**
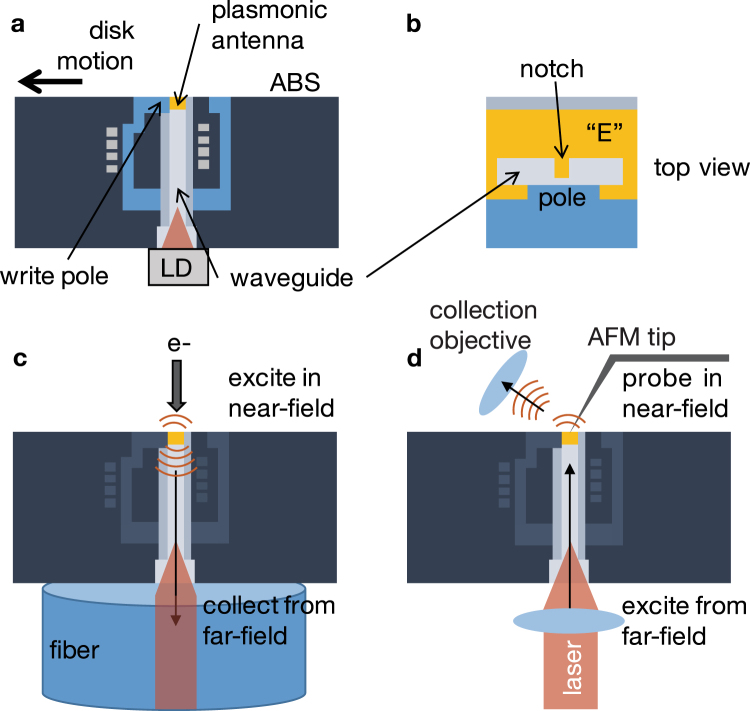
Schematics for bidirectional near-field characterization. (**a**) A side cross-sectional profile of a heat-assisted magnetic recording head (LD: laser diode, ABS: air-bearing surface), which incorporates a (**b**) waveguide-coupled plasmonic antenna surrounded by the standard magnetic write pole and coils. Bidirectional near-field characterization was performed using (**c**) scanning electron microscopy cathodoluminescence and (**d**) scattering scanning near-field optical microscopy. In **c**, the electrons in the near-field excite resonances in the antenna, which are coupled into the waveguide and collected in the far-field using a fiber. In (**d**) far-field laser light is focused into the head’s waveguide using the microscope’s objective, and the light is waveguide coupled into the plasmonic antenna whose near-field is probed using an AFM tip that scatters near-field light into the far-field where it is collected by another objective.

HAMR (Fig. 1a) has been identified as one of the best solutions to overcome the superparamagnetic limit, which dictates the magnetic media coercivity necessary to record thermally stable data “bits” of a given size for normal HDD operating conditions. The available write field strength limits storage density^3–5,7–9^. Smaller bits require magnetically “stiff” media whose polarization cannot be flipped with the magnet in a traditional perpendicular magnetic recording head without assistance. HAMR incorporates a plasmonic antenna that acts as a local heater of the magnetic media by converting energy from far-field laser light into near-field optical modes^3–6,9^. This high-gradient optical field heats a tight spot on the magnetic media close to its Curie temperature significantly reducing its coercivity and enabling its polarization to be written by the recording head. Since the drive’s performance and the size of the recorded bit are now determined by the plasmonic antenna (Fig. 1b), the success of the technology requires a thorough understanding of the antenna and waveguide coupling mechanism used for light delivery. Appropriate characterization techniques must be employed to investigate as-fabricated recording heads containing plasmonic antennas as well as their waveguide couplers. Scanning electron microscopy cathodoluminescence (SEM-CL, Fig. 1c) and scattering scanning near-field optical microscopy (sSNOM, Fig. 1d) were used to perform this bidirectional near-field characterization of waveguide-coupled plasmonic antennas in HAMR heads.

Cathodoluminescence (CL) occurs when electrons incident on a material cause the emission of photons through material-dependent coherent (plasmons) or incoherent (minority carrier generation and recombination, color center excitation) processes^10,11^. The resulting CL photons can be spatially mapped by SEM and spectroscopically analyzed with an attached spectrometer. Hyperspectral maps of the nanostructured optical response of the sample can be obtained with spatial resolution approaching 5–10 nm (*i*.*e*. the electron beam size/resolution of the SEM in use)^12^. SEM-CL has been used to generate plasmon resonance maps of metallic nanostructures^12–16^, and in this work it was used to probe the response of the waveguide-coupled plasmonic antenna incorporated into a prototype HAMR head. While the electrons have easy access to the plasmonic antenna located at the top of the head on the air-bearing surface (ABS), full characterization of the head’s optical system requires collection of the CL photons at the output of the head’s integrated waveguide.

**Figure 2:**
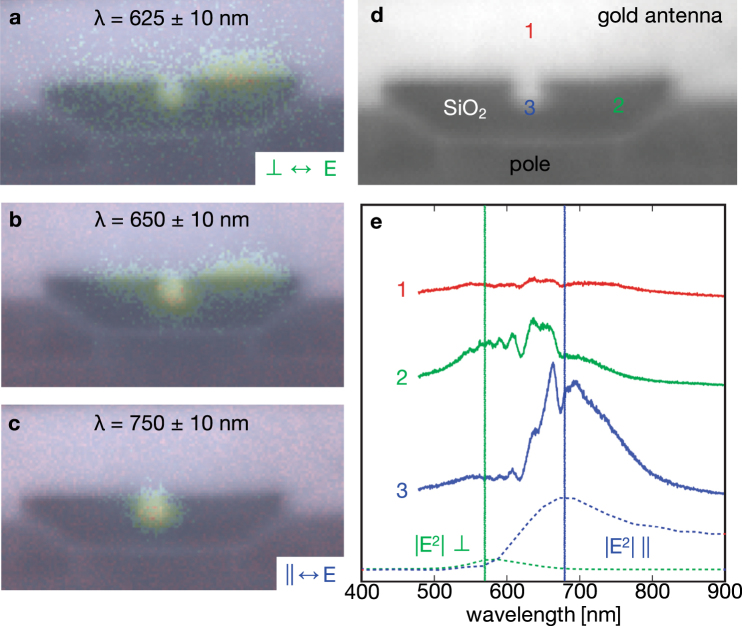
Cathodoluminescence analysis as performed inside a scanning electron microscope. (**a–c**) Full field intensity maps were collected over the antenna region for discrete and narrow (20 nm) wavelength windows revealing the spatial location of the resonances on the air-bearing surface. (Additional maps for other wavelengths can be found in the Supporting Information.) E-antenna notch width ~50 nm. Based on these maps, (**d**) three distinct and different points on the air-bearing surface surrounding the plasmonic antenna were chosen and (**e**) full measured spectra (solid lines) were compared with modeling (dashed lines).

Intensity mappings were generated by integrating the signal over 20 nm wavelength windows to better observe the head’s (antenna’s) resonant behavior as a function of output wavelength. Near 625 nm (Fig. 2a), the right side at the top metal face of the E-shaped antenna exhibits the strongest resonance. With increasing wavelength, a resonance begins to appear at the “notch” region (center of the “E”) as the side resonance begins to disappear (Figs 2b, S5). Near 750 nm (Fig. 2c), the output CL photons are localized to only the notch region.

Complete spectra shown in Fig. 2e were collected from three regions of interest marked in Fig. 2d: 1. Approximately 100 nm above the center of the “E” notch (a low CL intensity reference), 2. The region to the right of the notch overlapping with the waveguide (resonant near 625 nm), and 3. The notch region, which localizes the optical field for heating the magnetic media (resonant near 665–760 nm). The antenna is designed to be resonant at 830 nm during drive operation, but this resonance is blue-shifted when no magnetic media is present. Features in the spectra (Fig. 2e) can be attributed to three sources. First, the plasmonic resonance is evident as the broad feature matching the modeled profiles. Second, the sharp peaks near 650 nm are due to the well-known luminescence of oxygen-rich SiO_2_^17^. Finally, the lower wavelength bumps are likely due to wavelength-dependent cavity effects from the waveguide itself. The results of wavelength and polarization-dependent electromagnetic simulations of field intensity at the disk height when the antenna is excited through the integrated waveguide are shown for region (3) at the bottom of Fig. 2e. The waveguide-antenna system was engineered to focus parallel-polarized light (relative to the notch) to position (3) at resonance, while perpendicular-polarized light is neither focused nor resonantly enhanced at the notch region but does exhibit a delocalized mode spread throughout the stem of the E^18,19^. The CL collection system using the multimode fiber did not preserve the polarization of the transmitted light, but it is believed that the general agreement between the shape of the CL spectra in region (3) with the parallel simulation is consistent with CL excitation of the plasmonic resonance. The spectra of region (2) may be due to excitation of the delocalized perpendicular waveguide mode combined with luminescence from the SiO_2_, while region (1) does not couple well to the waveguide modes. The experimental system could be improved through the use of a polarization-maintaining optical fiber for collection.

**Figure 3:**
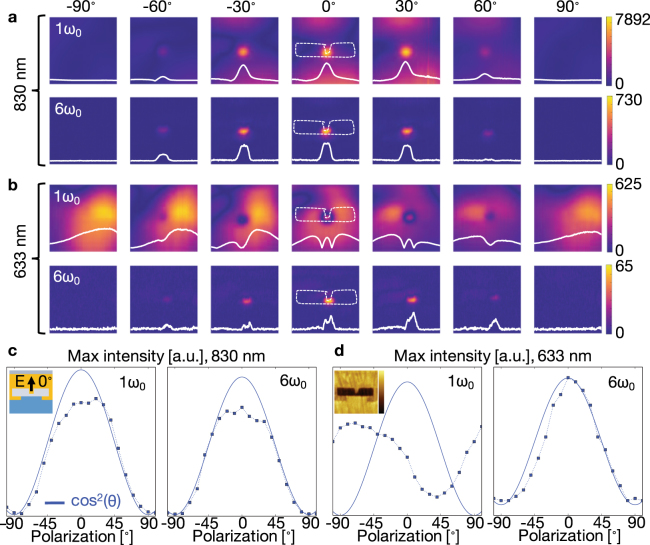
Scattering scanning near-field optical microscopy images of HAMR heads as a function of wavelength and polarization. Near-field maps for the 1ω_0_ and 6ω_0_ with both (**a**) 830 nm and (**b**) 633 nm wavelengths as well as polarizations ranging from −90° deg (perp, TE) to 0° (para TM) to +90° (perp, TE). All maps are 400 nm × 400 nm. The intensity maxima from all maps were extracted and plotted against the expected cos^2^(*θ*) intensity decay curve for both (**c**) 830 nm light and (**d**) 633 nm light. The full data set ranged from −100° to 100° in increments of 10° and covered six harmonics for both wavelengths. The AFM color scale ranges from −3.8 to +1.6 nm, and the map is 400 nm × 400 nm. Additional images can be found in the Supporting Information.

sSNOM was used to directly map the near-field intensity distribution of the HAMR device close to the ABS as a function of the waveguide excitation wavelength and polarization. sSNOM uses an atomic force microscopy (AFM) tip as a local scattering center, coupling near-field light into the far-field where it can be collected by an objective for detection^20–23^. With the use of a lock-in amplifier to record different harmonics of an AFM tip oscillating at the cantilever resonant frequency (ω_0_), the desired near-field signal can be separated from the background far-field light with good signal-to-background sensitivity^24,25^. sSNOM is an excellent technique for probing plasmonic structures^26–30^, and was used to characterize the behavior of a HAMR waveguide-coupled plasmonic antenna both in this work as well in our previous work^9,31^. In these sSNOM experiments, external laser light was coupled into the integrated HAMR waveguide exciting the plasmonic antenna much like in drive operation. As the sample scanned under the oscillating tip, the different harmonic components of the scattered signal were collected and mapped. As reported previously, the higher harmonics (5ω_0_ − 6ω_0_) were more representative of the near-field profile as seen by the magnetic disk during drive operation. Near-field maps were collected for six harmonics over a range of incident laser polarizations (−100° to 100° in increments of 10° where 0° was parallel to the notch) for incident wavelengths of 830 nm (Fig. 3a), the designed head operation wavelength, as well as 633 nm (Fig. 3b) in order to more fully map out the HAMR performance and to complement the SEM-CL measurements.

Several observations can be made from the data in Fig. 3. The harmonics’ intensity for the 830 nm light is >10× that of the 633 nm light, which was expected since the head (waveguide and plasmonic antenna) was designed to operate at 830 nm. At this wavelength, the polarization dependence of “nanofocusing” was observed for all harmonics (Figs 3a,c, S7–9), with the expected maximum intensity for parallel (0°) excitation and minimum intensity for perpendicular (−90° and 90°) excitation^18,19^. The observed maximum intensities deviated from the expected cos^2^(*θ*) dependence on polarization angle *θ* near the parallel polarization^1^. It is believed that this was an experimental artifact due to saturation of the avalanche photodiode (APD) or the lock-in preamplifier due to the large far-field transmission of the waveguide at resonance. The waveguide was designed to operate most efficiently with parallel polarization at 830 nm, and only a fraction of the laser light is converted to the evanescent near-field by the plasmonic antenna. It is worth noting that follow-up measurements of the 0° parallel polarization were performed with care to eliminate saturation. Similar decay trends in maximum intensity as a function of harmonic were observed when compared to the saturated set presented here confirming the cause of the saturation, demonstrating its spatially uniform nature, and validating the presented near-field mappings.

The background saturation observed in the 830 nm data set was not observed in the data set collected using 633 nm laser light as is evident in Fig. 3d right in which the 6ω_0_ nicely follows the expected cos^2^(*θ*) decay trend. In addition, the presence of a near-field resonance of smaller intensity for the parallel (0°) electric field matched the results from the SEM-CL measurements and modeling. The fundamental frequency’s (1ω_0_) harmonic mappings also show the same behavior as observed with SEM-CL; with perpendicular electric field polarizations (−90° and 90°), the recorded intensity of the resonance was dominant on the right of the E-shaped antenna’s notch feature. Comparing the sSNOM near-field mappings in Fig. 3b, 1ω_0_ with the SEM-CL map in Fig. 2b centered at 650 nm suggests that this wavelength region of SEM-CL data is largely comprised of CL photons with a variety of polarizations, especially when considering the presence of a smaller relative intensity feature on the left side of the notch as well as the reduced intensity of the rightmost feature relative to the intensity maximum located at the notch. Since the fundamental frequency’s (1ω_0_) scattering cross-section is known to couple more light from the optical far-field, and these side resonances are not present in the higher harmonics (6ω_0_), we can conclude that the corresponding SEM-CL signal (625–675 nm) is more likely due to optical excitations that couple well into the far-field modes of the waveguide-plasmonic antenna system. These characterizations of the near- versus far-field and polarization components for the different features observed in the SEM-CL and sSNOM maps are also consistent with the simulated spectra for the antenna. The complete set of near-field mappings for all measured polarizations, wavelengths, and six harmonics as well as their maximum intensity curves can be found in the Supplemental Information.

In conclusion, this study reports the first visualization of the optical transfer function for near-field enhancement in waveguide-coupled plasmonic antennas using both near-field excitation mapping and probed near-field mapping by SEM-CL and sSNOM. Both techniques were able to provide unique insight into the HAMR head’s behavior during drive operation and conclusions drawn from their results exhibited strong agreement. Furthermore, the usefulness of the described methods has been firmly demonstrated for the development of HAMR technology as well as future devices based on the near-field properties of light.

## Methods

SEM-CL measurements were performed at the Molecular Foundry using custom instrumentation with the Zeiss Supra 55VP FE-SEM with an accelerating voltage of 30 kV. Samples containing multiple HAMR heads were mounted above an optical collection fiber in a custom holder (see Supplementary Information for details). A HAMR “rowbar” (a linear array of fabricated HAMR heads not yet diced into individual sensors) was mounted on an XY nano-positioning stage (stacked Attocube ECSx3030 closed-loop high vacuum translators with better than 50 nm reproducibility), which was in turn mounted onto the SEM stepper-motor XYZ stage. A cleaved and polished multimode optical fiber (Thorlabs FG200LCC or equivalent, low-OH 200 um 0.22 NA) was supported from the SEM stage facing the SEM optics. The SEM XYZ stage was used to position the fiber in the center of the SEM image, next the nano-positioning stage inserted the HAMR rowbar sample so that a single sensor was centered in the SEM image.

The SEM electron beam was scanned over the surface of the HAMR plasmonic antenna region to excite near-field optical modes, which coupled through the integrated optical waveguide (used for excitation in HAMR applications). The CL light was collected by the proximity-coupled optical fiber and delivered to external collection optics. The CL photons output from the fiber were then coupled either to a spectrometer and CCD camera or to a photon-counting APD with a band-pass filter as the electrons were raster scanned over the antenna region. A custom vacuum feedthrough was fabricated from a 1/8” Swagelock UltraTorr fitting. Collection fibers had an SMA connector at the external end, and were epoxied into a short section of 1/8” OD polished stainless steel tubing, which was inserted through the UltraTorr fitting, allowing a compression seal to be made on the steel tube.

Optical CL spectroscopy was performed using an Acton 2300i spectrograph with a 150 line/mm grating blazed at 500 nm coupled to an Andor Newton EMCCD spectroscopic camera, both controlled by custom LabView software. An input coupling lens matched the 0.22 fiber NA (numerical aperture) to that of the spectrometer (0.1 NA or f/4). Optical CL images/maps were collected using 20 nm band-pass optical filters and a Perkin-Elmer APD (avalanche photo-diode) single-photon detector. A Nikon 10× objective coupled the filtered fiber output into the APD.

The modeling work was performed using Lumerical FDTD Solutions, and the integrated intensity spectra were extracted to generate the dashed signal in Fig. 2e.

sSNOM measurements were performed at HGST with the AIST-NT CombiScope 1000-SPM and tips from Nanosensors (ATEC-NC). The auto non-contact/tapping default mode was used with the 160 μm cantilevers (~300 kHz resonance), 80 nm oscillation amplitude, and 87% set point. An 830 nm laser (diode: Sanyo DL8142–201) and a 633 nm HeNe laser (JDSU 1145 P) were each separately polarized (Thorlabs LPVIS100-MP), reduced to ~15 mW (continuous wave), and directed with free space optics into the microscope. The microscope’s bottom objective (Olympus ULWD MSPlan 50) mount was piezo tuned in three dimensions first for coarse alignment and then for optimal coupling of the laser spot into the waveguide once the tip was hovered over the plasmonic antenna. A 50× objective (Mitutoyo NIR M Plan Apo NIR 50× , 378-825-5) was precisely aligned with piezo scanners and used to collect the scattered light, which was then observed by an APD (Thorlabs APD120A). The resulting signal passed through a lock-in amplifier (Zurich Instruments HF2LI), and data were collected through the AIST-NT software.

### One Sentence Summary

Visualizations of the near-field modes in the region of a plasmonic device were created using scattering scanning near-field optical microscopy and scanning electron microscopy cathodoluminescence with both showing a strong correspondence to multiphysical numerical modeling of the devices under interrogation.

## Electronic supplementary material

Supplementary Information
