# Supplementary material for: Visualizing the bidirectional optical transfer function for near-field enhancement in waveguide coupled plasmonic transducers
Source: Sci Rep. 2018 Apr 10;8:5761. doi: 10.1038/s41598-018-24061-3 (PMC5893547; doi:10.1038/s41598-018-24061-3)

Supplementary information for:

Visualizing the bidirectional optical transfer function for near-field enhancement in waveguide coupled plasmonic transducers

Lauren M. Otto[1,2], D. Frank Ogletree[3], Shaul Aloni[3], Matteo Staffaroni[2], Barry C. Stipe[2], Aeron T. Hammack*[,2,3]

[1] Electrical and Computer Engineering, University of Minnesota, Minneapolis, MN, USA

[2] HGST (Western Digital Corporation) San Jose & Fremont, CA, USA

[3] Molecular Foundry, Lawrence Berkeley National Laboratory, Berkeley CA, USA

[*] Address correspondence to: aeronth@berkeley.edu

This supplementary information contains schematics and full data sets for the two techniques discussed in the main text.

Figure S1: Schematics comparing hard disk drive write head profiles. a. for standard perpendicular magnetic recording and **b.** for next-generation heat-assisted magnetic recording, which incorporates many additional elements including a waveguide-coupled plasmonic antenna.

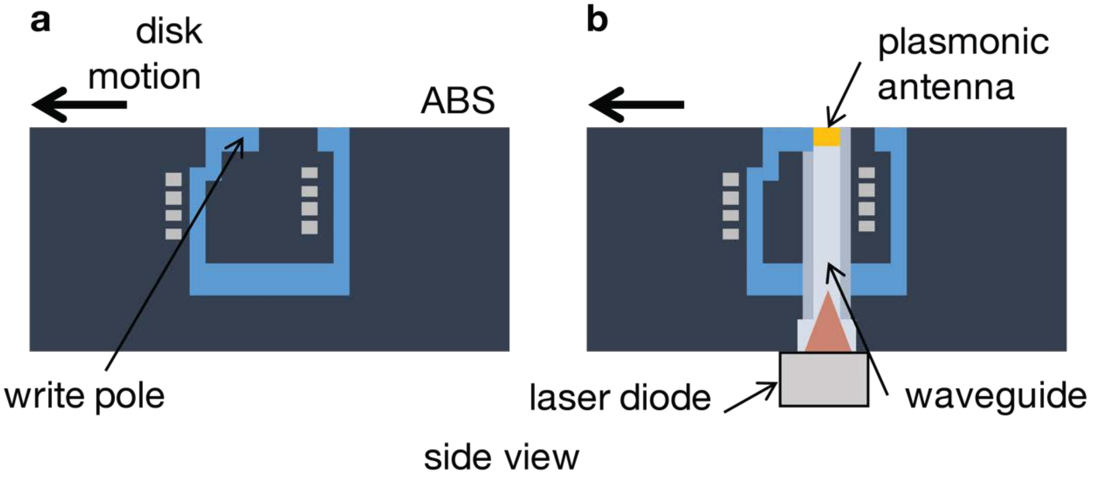

I. SEM-CL

Figure S2: Large-scale scanning electron microscopy (SEM) image of a heat-assisted magnetic recording (HAMR) head mounted on an optical fiber for cathodoluminescence (CL) imaging.

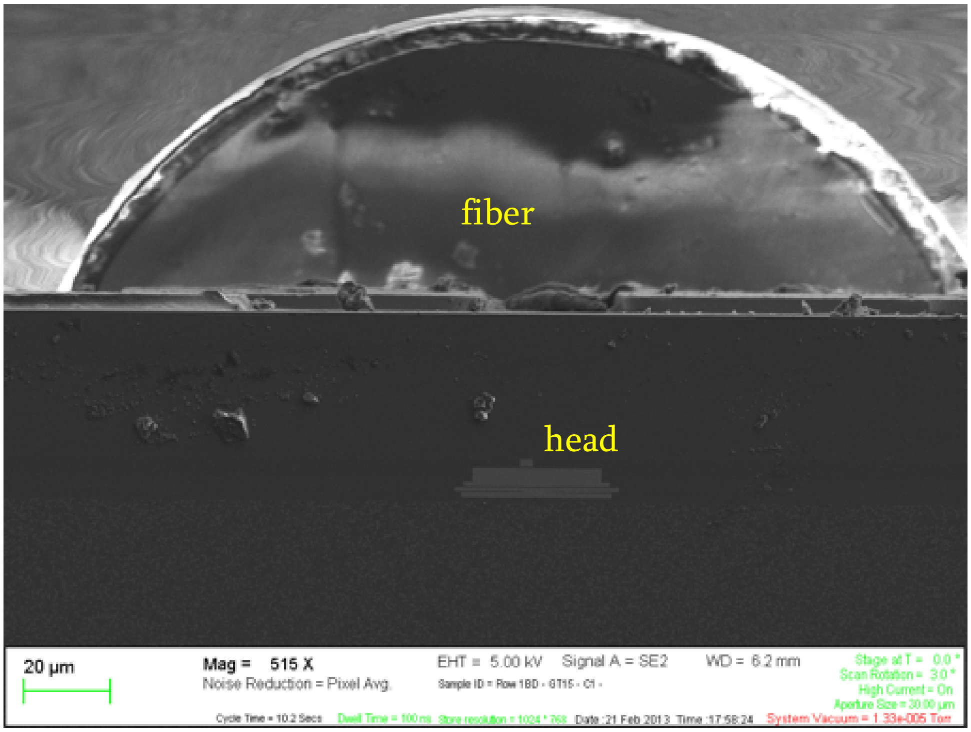

Figure S3: Mounting schematic for SEM-CL experiments. An entire rowbar (a set of many of many HAMR heads not yet diced post fabrication) was mounted into the SEM holder.

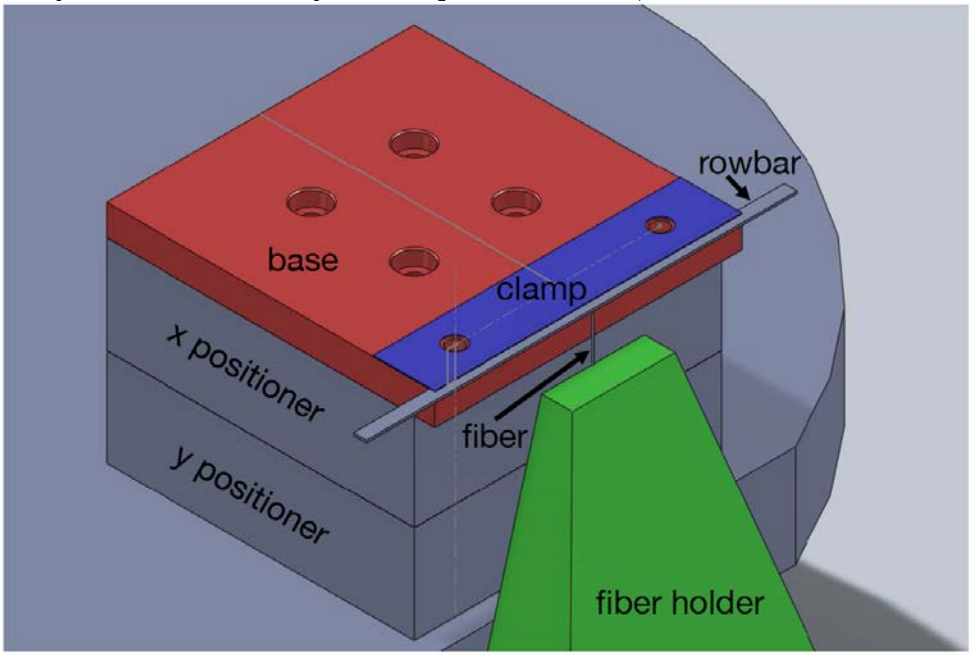

Figure S4: Detection schematic of the SEM-CL signal. Detection employed a photomultiplier tube (PMT) for spectral imaging, or an electron-multiplying charge-coupled device (EMCCD) for spectra, following dispersion through an imaging spectrometer.

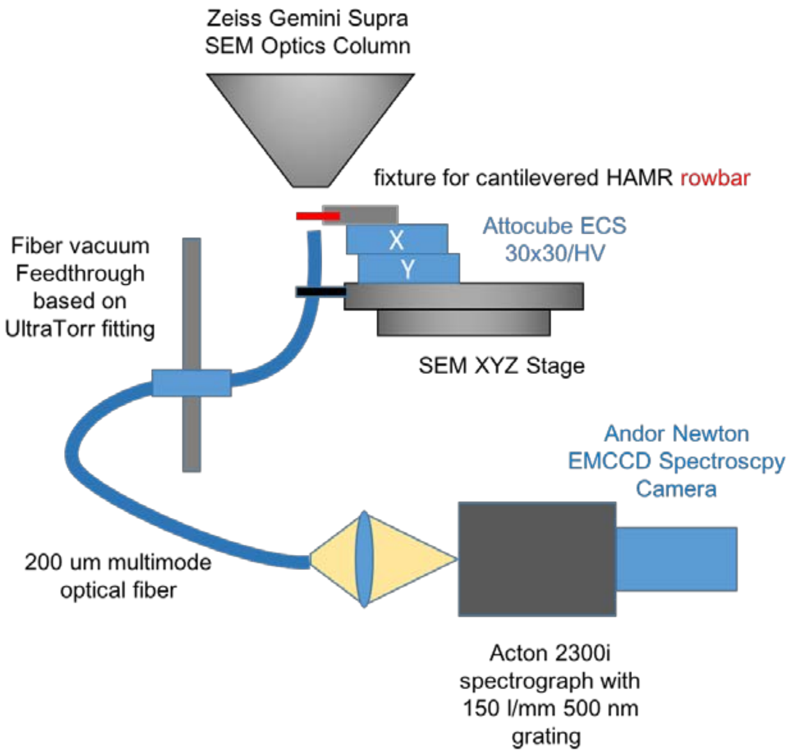

Figure S5: The complete set of recorded SEM-CL mappings over 20 nm wavelength windows as in Figure 2.

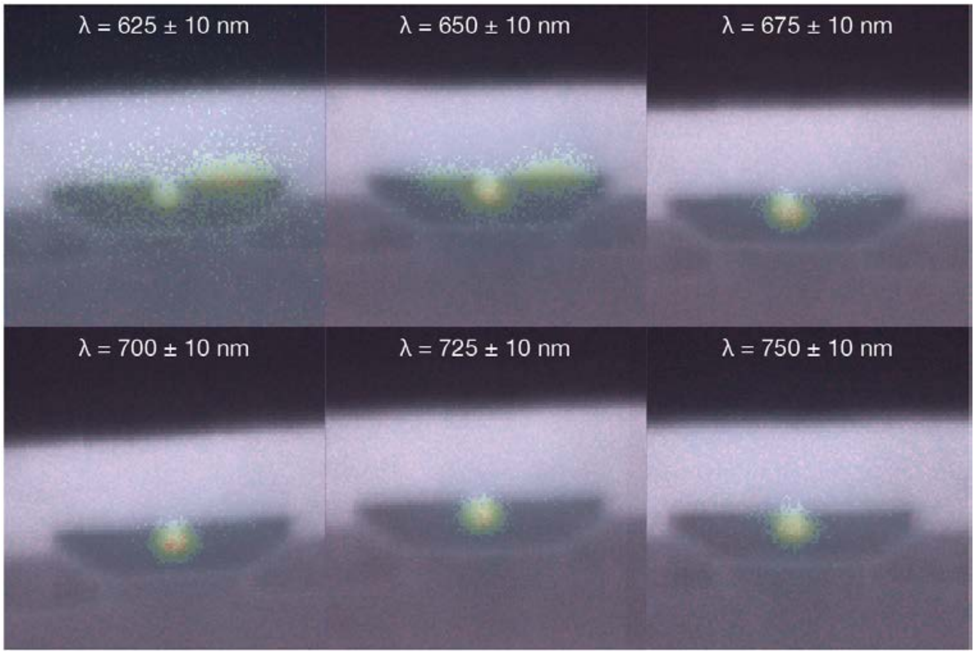

II. sSNOM

Figure S6: Schematic of the heat-assisted magnetic recording (HAMR) heads as imaged by the AIST-NT sSNOM system. A side profile of **a.** a HAMR head and **b.** the sSNOM system, including the layout of the scanned surface of the HAMR head. **c.** Camera views from the side (left) and bottom (right) objectives of the sSNOM system. When the AFM tip was positioned over the antenna, each objective would be piezo scanned to allow for precise alignment of the laser light through the waveguide and to the antenna. Images would be collected from the objective scans to determine the precise position necessary for optimal alignment.

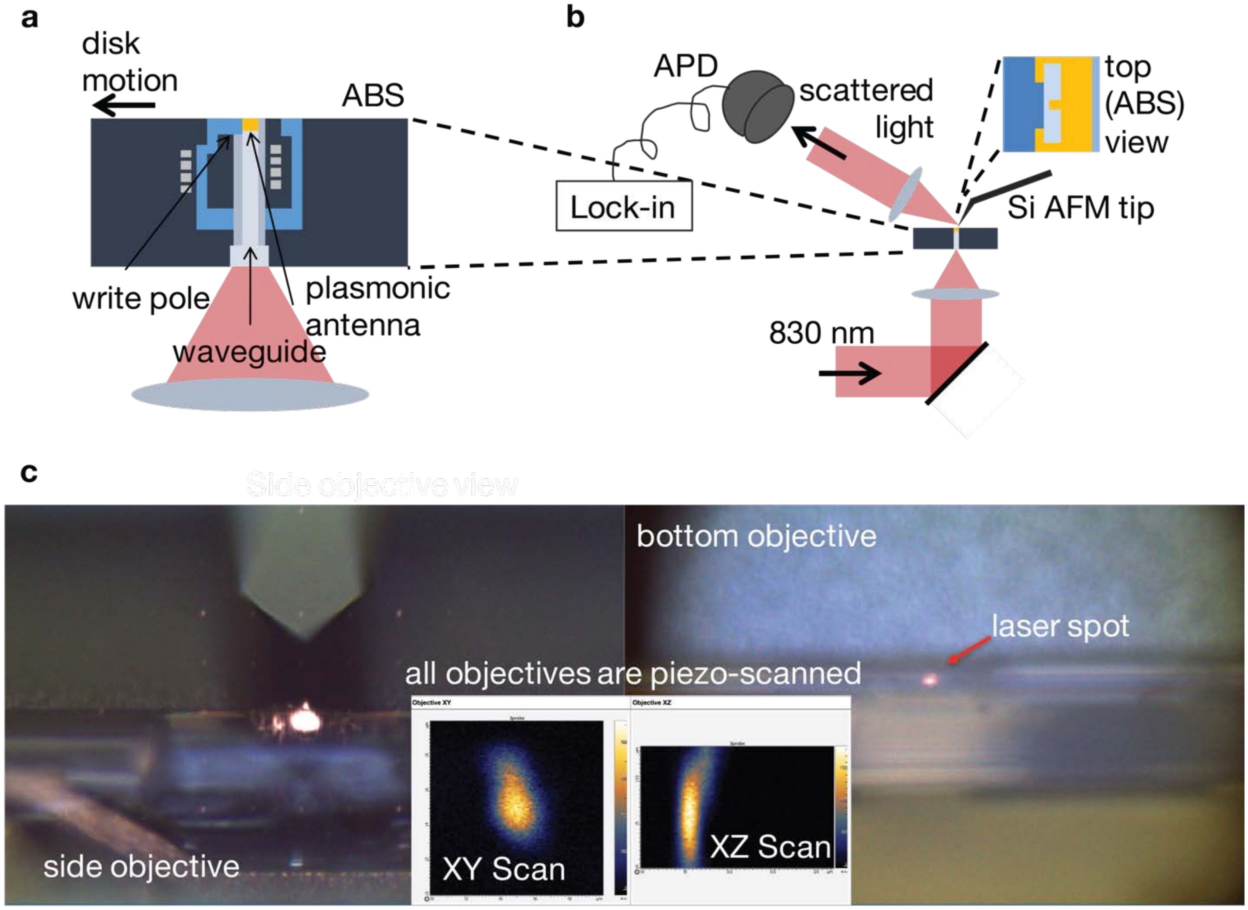

A. 830 nm laser excitation

Figure S7: The full set of sSNOM data taken with 830 nm laser light with harmonics $1\omega_0$ to $6\omega_0$ and polarizations from -100° to +100°.

$1\omega_0$

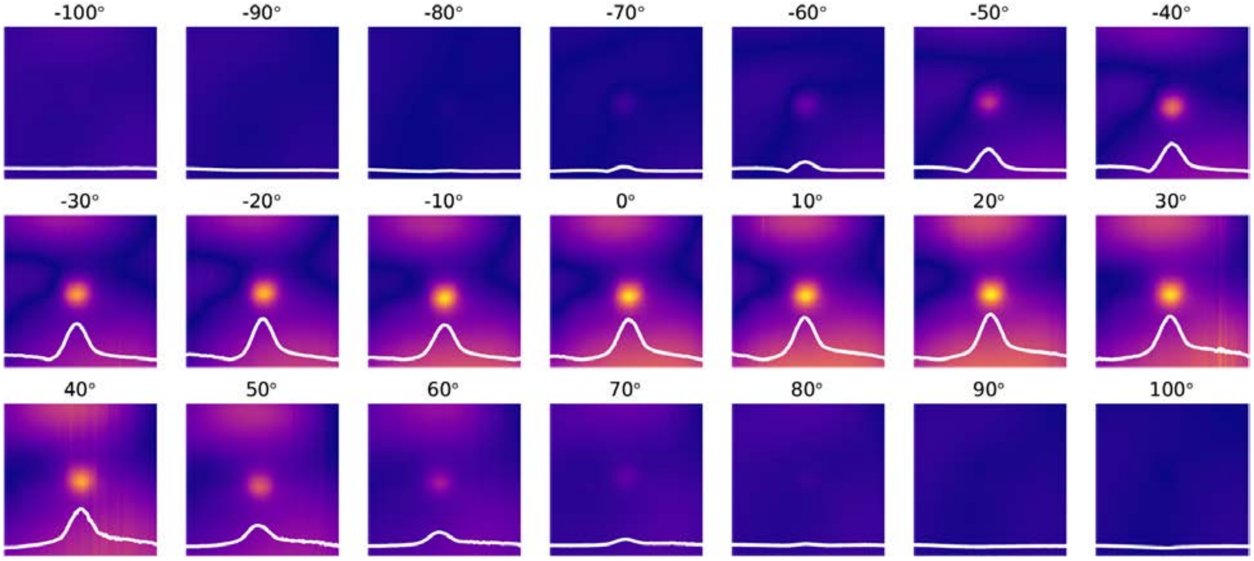

$2\omega_0$

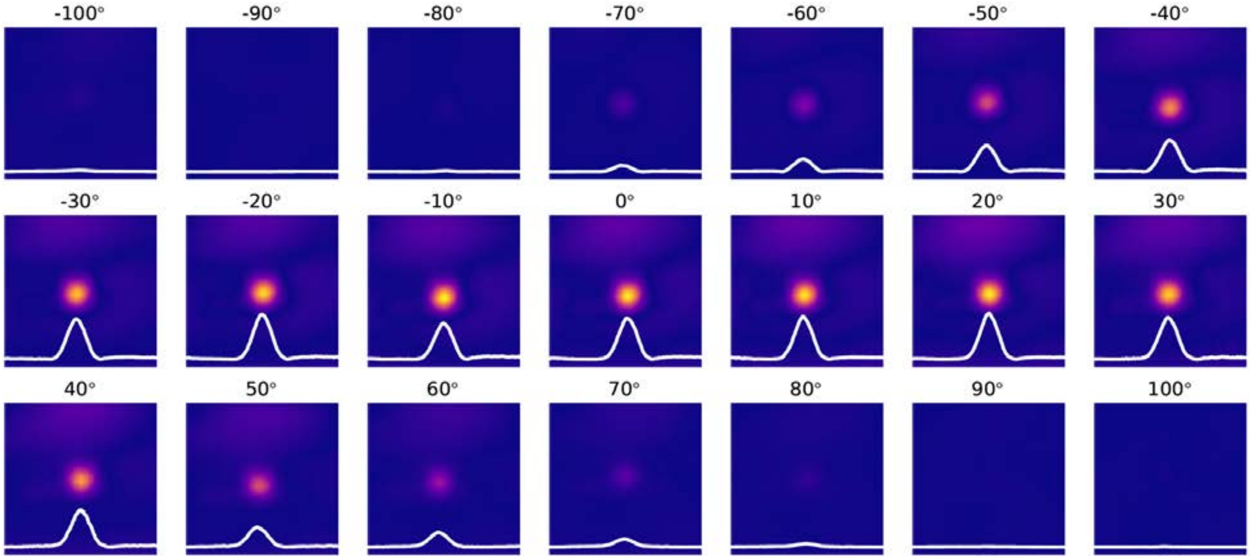

$3\omega_0$

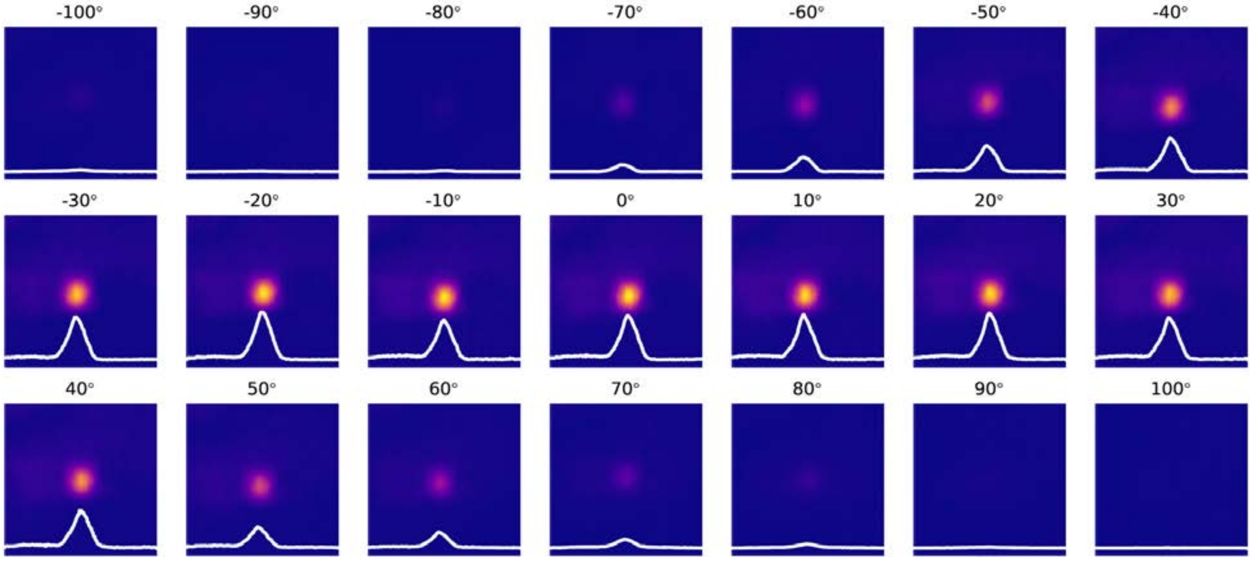

$4\omega_0$

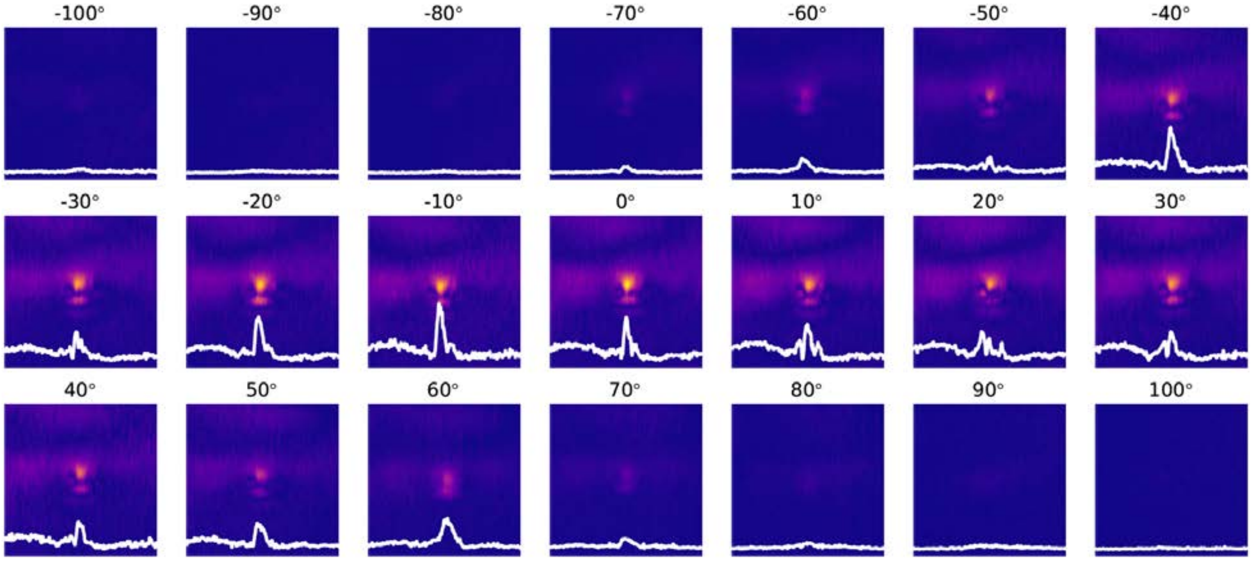

$5\omega_0$

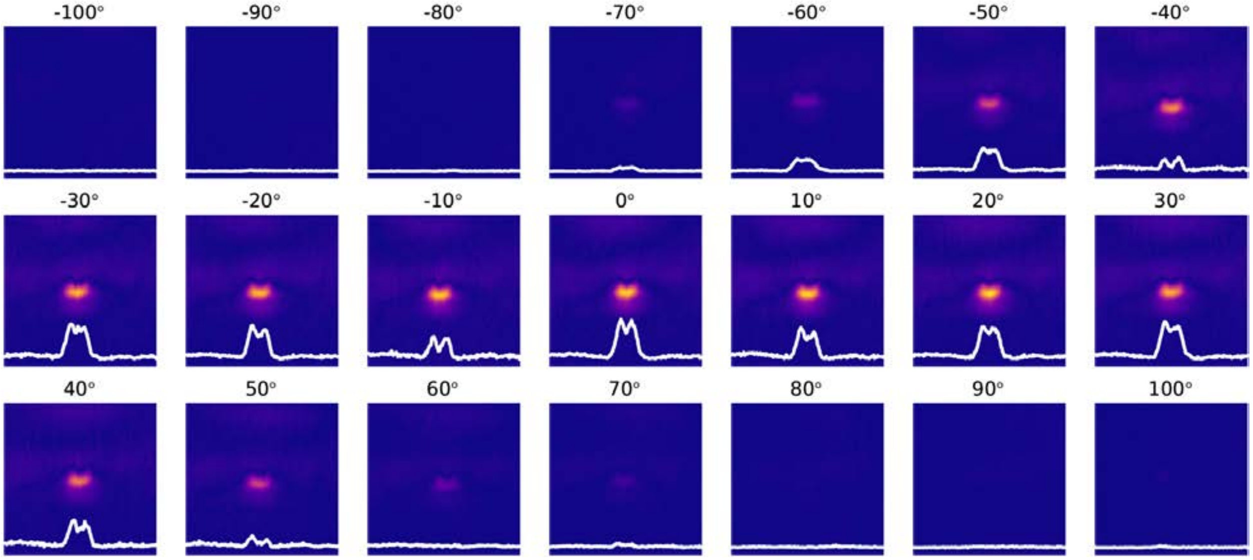

$6\omega_0$

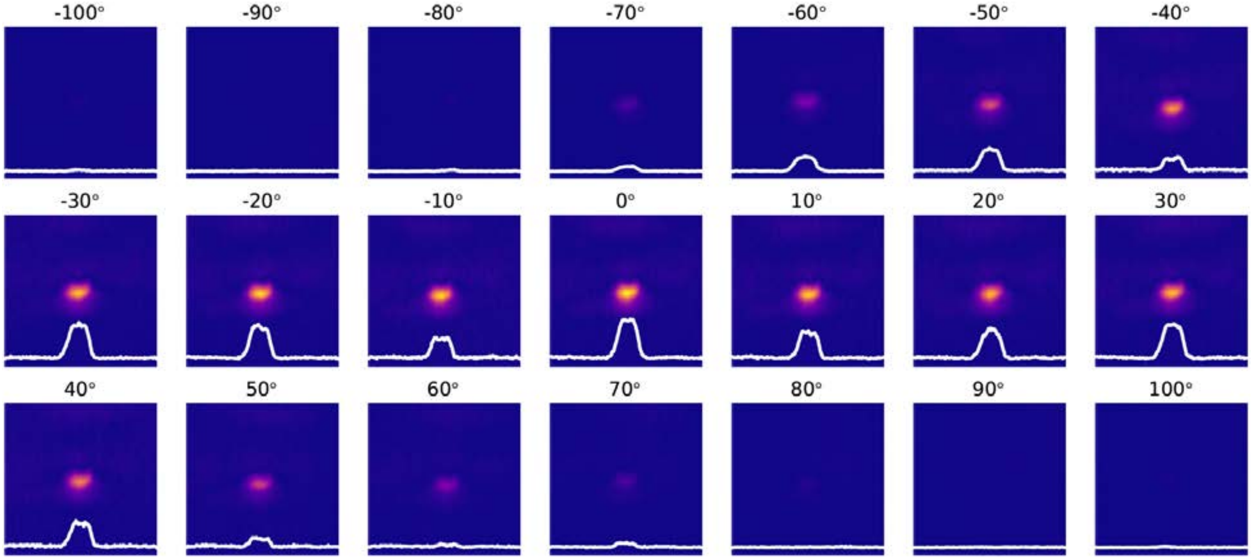

Figure S8: Maximum intensities of all the near-field maps in Figure S6. The data are plotted along with an expected intensity curve following a $\cos^2(\theta)$ law.

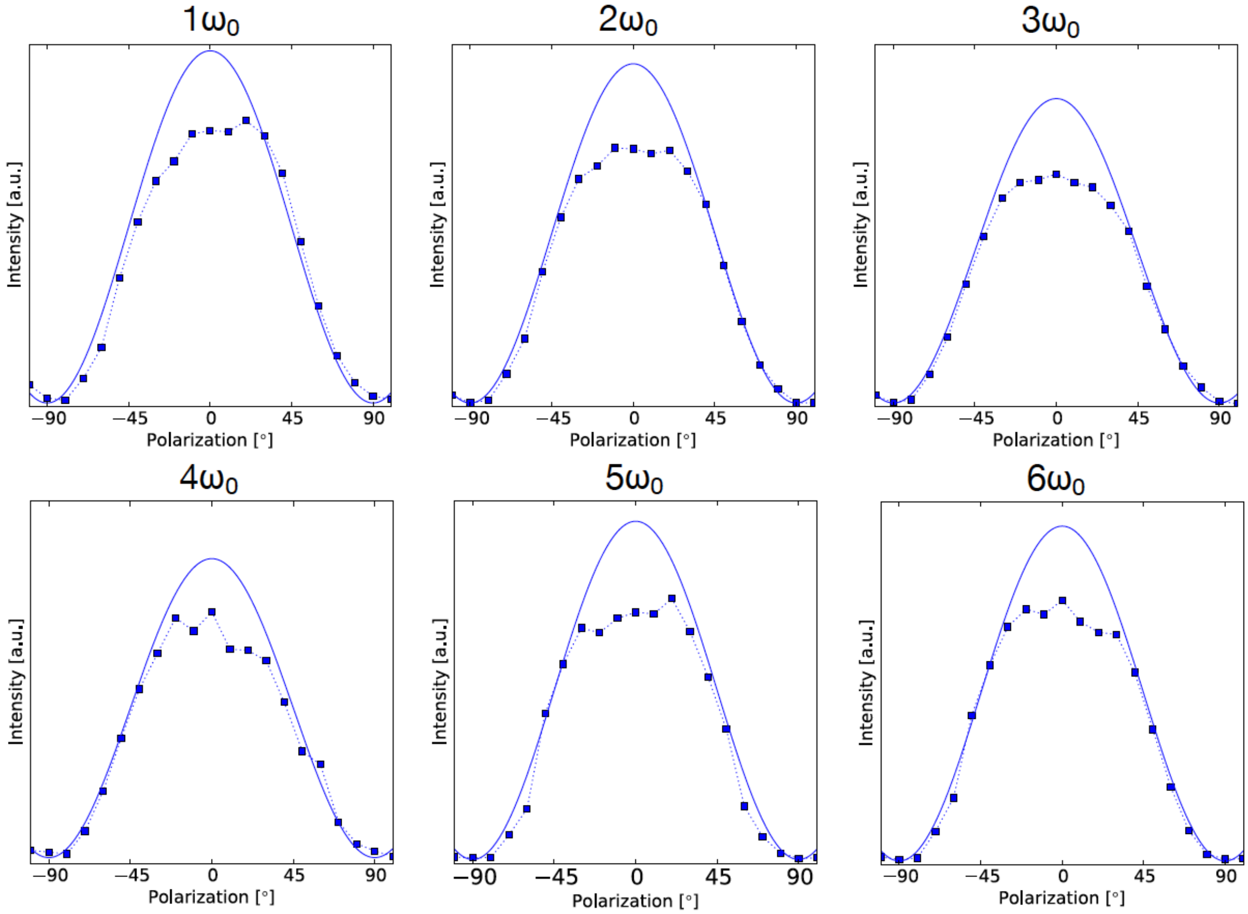

Figure S9: Maximum intensity vs. harmonic at 0° polarization with 830 nm laser excitation.

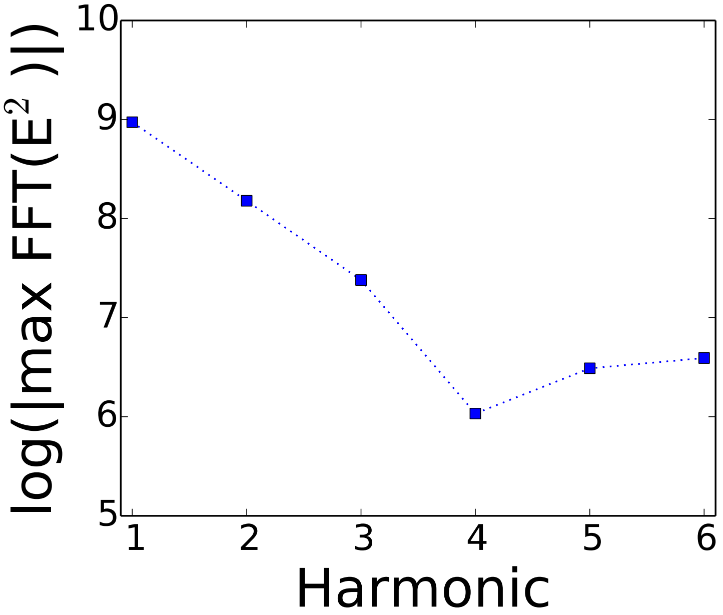

B. 633 nm laser excitation

Figure S10: The full set of sSNOM data taken with 633 nm laser light with harmonics $1\omega_0$ to $6\omega_0$ and polarizations from -100° to +100°.

$1\omega_0$

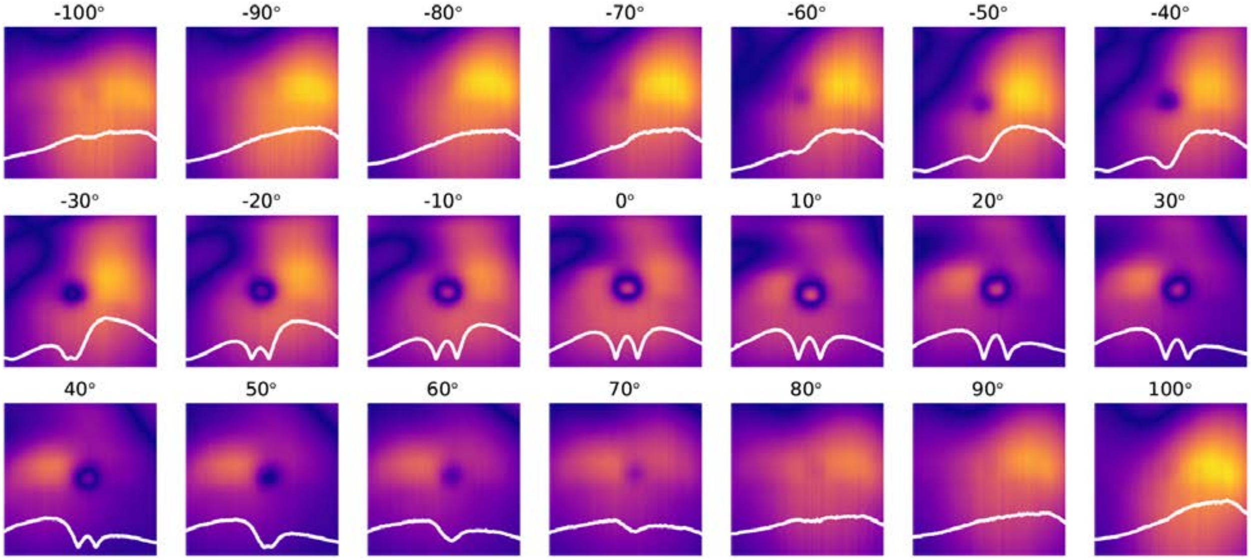

$2\omega_0$

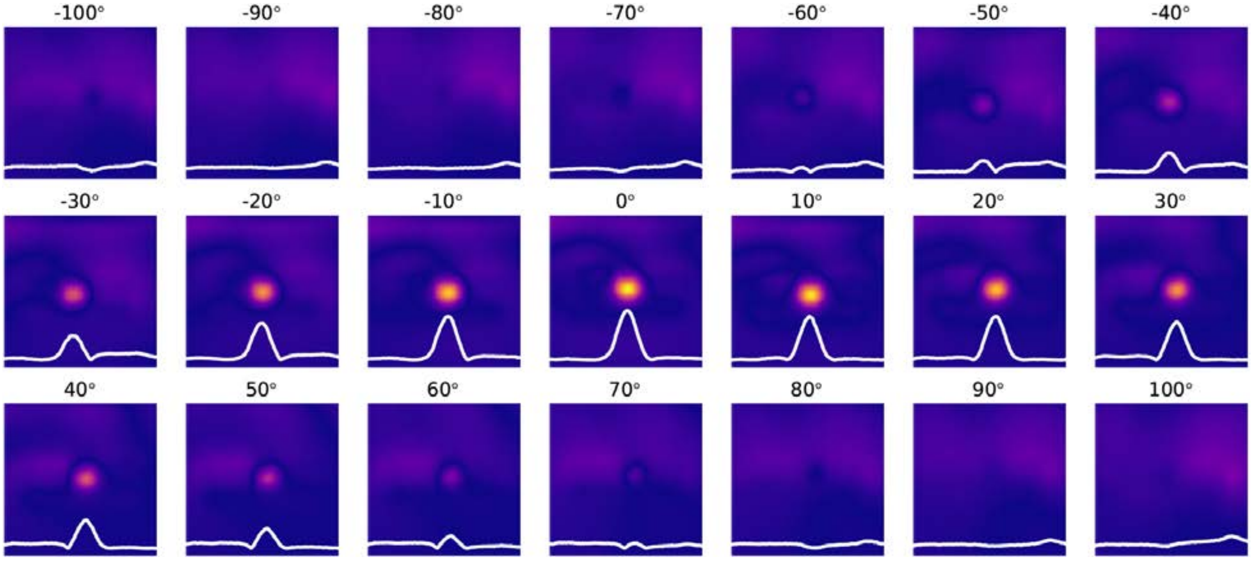

$3\omega_0$

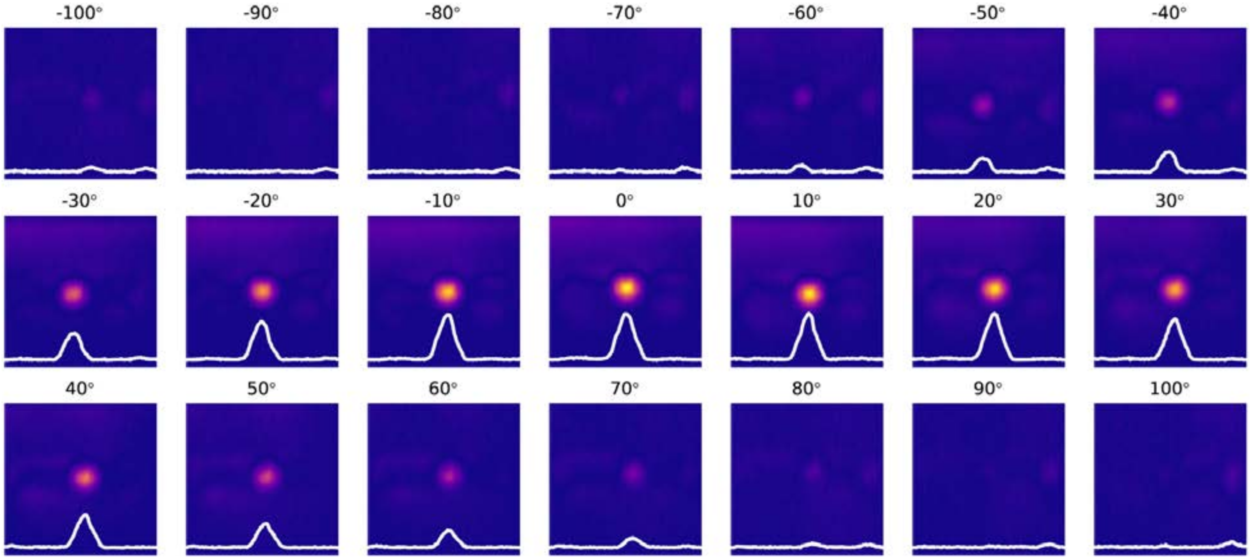

$4\omega_0$

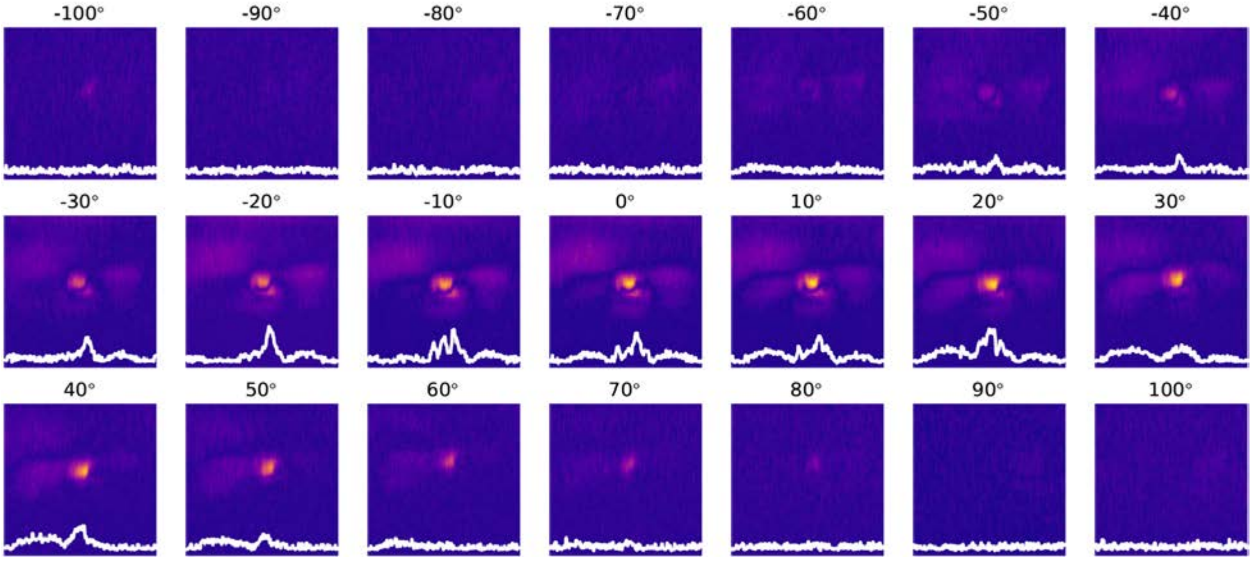

$5\omega_0$

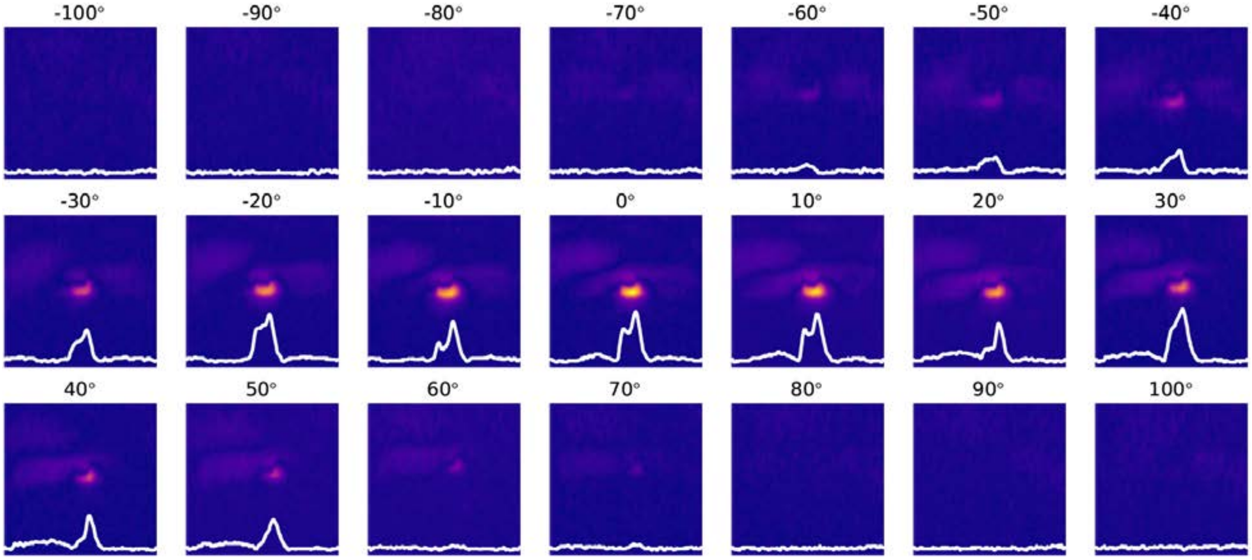

$6\omega_0$

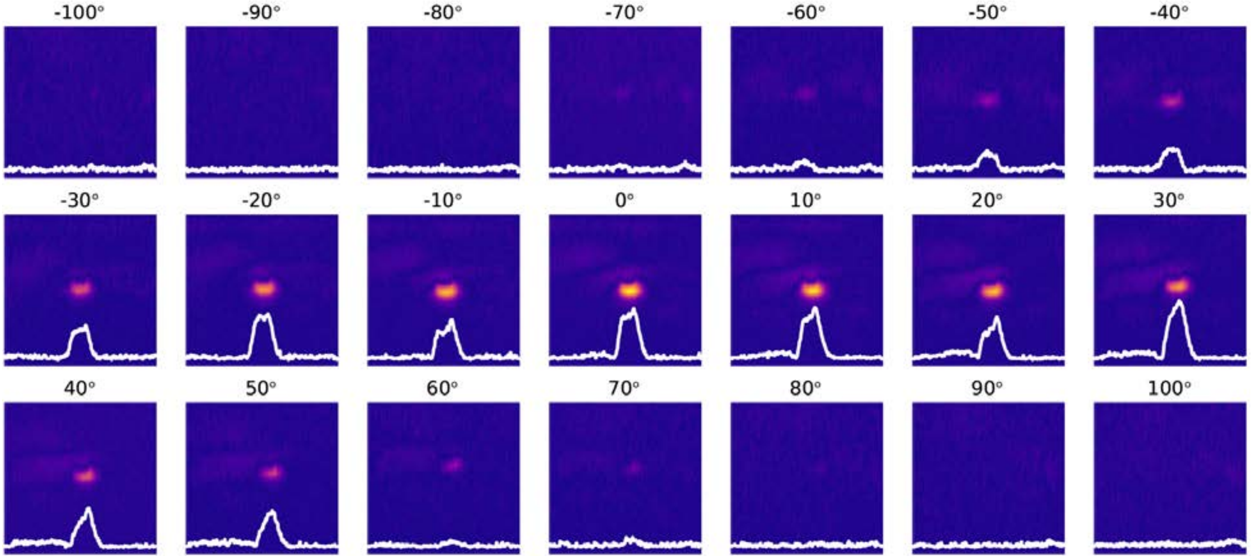

Figure S11: Maximum intensities of all the near-field maps in Figure S9. The data are plotted along with an expected intensity curve following a $\cos^2(\theta)$ law.

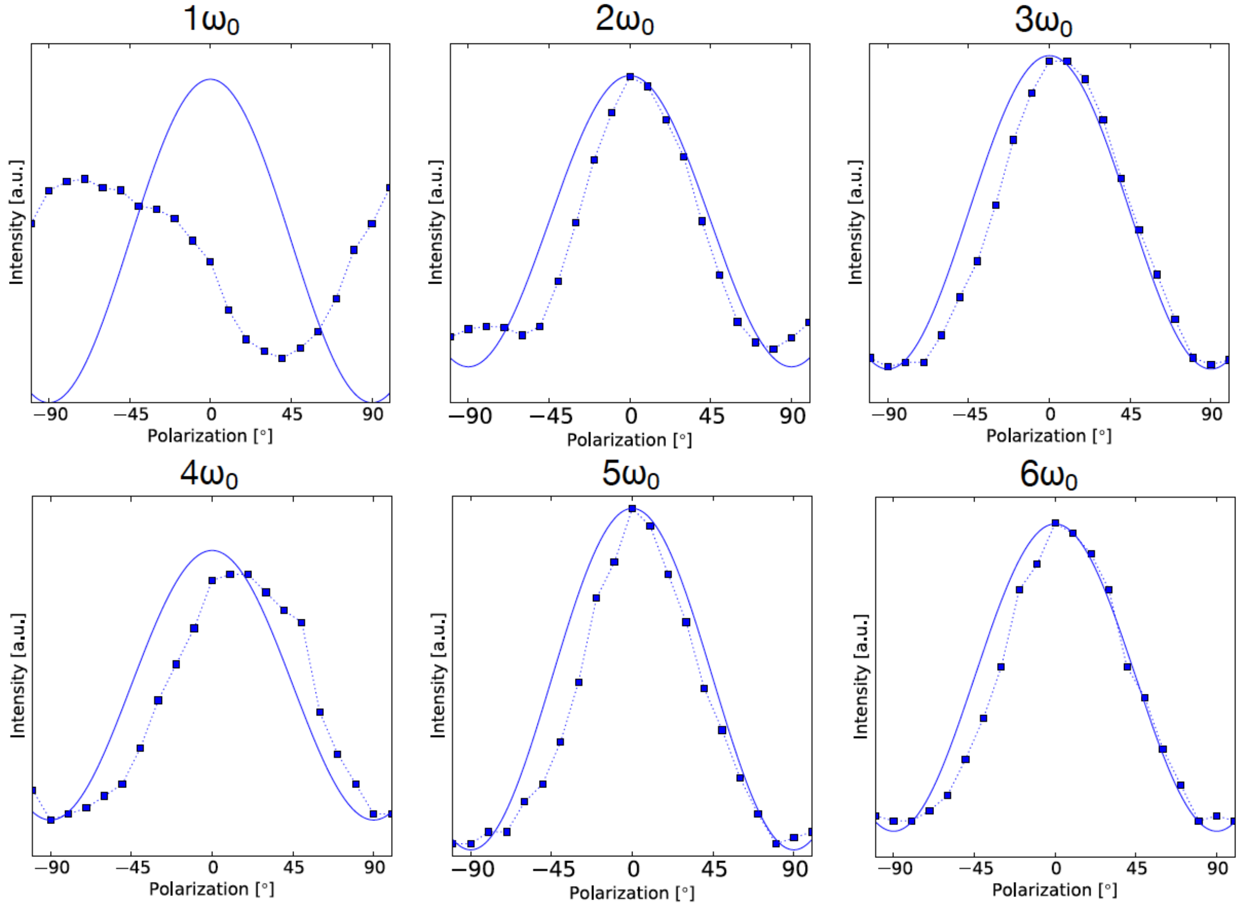

Figure S12: Maximum intensity vs. harmonic at 0° polarization with 633 nm laser excitation.

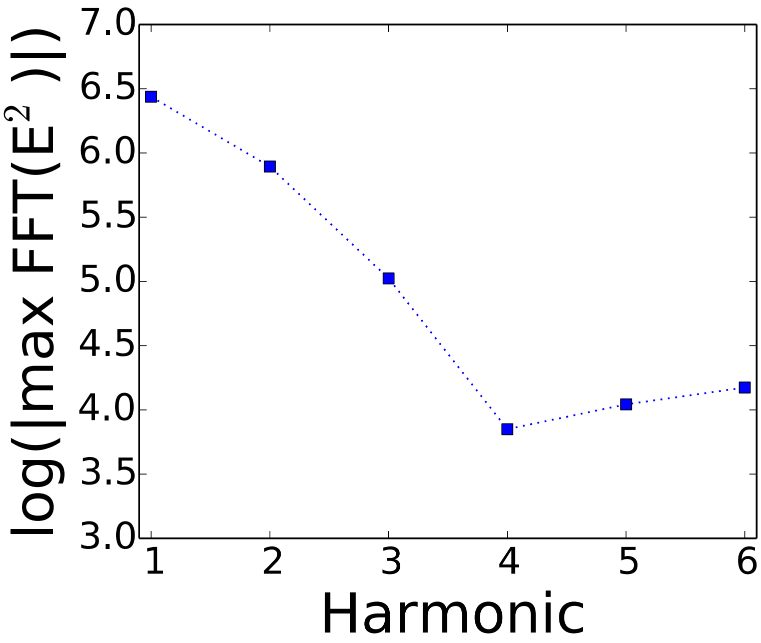

Supplement: Supplementary file 1 — Supplementary Information [file 41598_2018_24061_MOESM1_ESM.pdf]
